# Influence of Salinity, Temperature, Photoperiod, and *Isochrysis galbana* Microalgal Cell Density on the Growth of the Marine Copepod *Oithona nana*

**DOI:** 10.3390/ani15172635

**Published:** 2025-09-08

**Authors:** Jordan I. Huanacuni, Margaret Jennifer Nieto-Rojas, Renzo Pepe-Victoriano, Juan Zenón Resurrección-Huertas, Luis Antonio Espinoza-Ramos

**Affiliations:** 1 Grupo de Investigación Acuicultura Sostenible, Facultad de Ciencias Agropecuarias, Universidad Nacional Jorge Basadre Grohmann, Tacna 23000, Peru; jordan.92ihp@gmail.com; 2 Núcleo de Investigación Aplicada e Innovación en Ciencias Biológicas, Facultad de Recursos Naturales Renovables, Universidad Arturo Prat, Arica 1000000, Chile; 3 Piscigranja Tacna Bonita, Finfish Aquaculture Sociedad Anónima Cerrada, Tacna 23004, Peru; 4 Facultad de Ingeniería Pesquera, Universidad Nacional Agraria La Molina, Lima 15024, Peru; jenni94mnr@gmail.com; 5 Área de Biología Marina y Acuicultura, Facultad de Recursos Naturales Renovables, Universidad Arturo Prat, Iquique 1110000, Chile; 6 Escuela Profesional de Ingeniería Acuícola, Facultad de Ingeniería Pesquera, Universidad Nacional José Faustino Sánchez Carrión, Huacho 15138, Peru; pezsaulo@gmail.com; 7 Escuela Profesional de Ingeniería Pesquera, Facultad de Ciencias Agropecuarias, Universidad Nacional Jorge Basadre Grohmann, Tacna 23000, Peru

**Keywords:** culture parameters, live food, marine aquaculture, phytoplankton, zooplankton

## Abstract

Copepods play a critical role in aquatic food chains and are a key source of food in aquaculture, so optimizing their culture conditions is essential for their large-scale production. The objective of this study was to determine the optimal culture parameters of the copepod *Oithona nana* as a function of total population growth and the different stages of its life cycle. To this end, four experiments were carried out in quadruplicate, evaluating the effects of temperature, salinity, photoperiod and optimal microalgal concentration of *Isochrysis galbana*. Each experiment lasted 15 days. Temperatures of 20, 24, 28 and 32 °C were tested; salinity levels of 20, 25, 30 and 35 PSU; photoperiods of 12L:12D, 16L:8D and 24L:0D; and concentrations of *I. galbana* of 1 × 10^4^, 5 × 10^4^, 10 × 10^4^, 15 × 10^4^ and 20 × 10^4^ cells/mL. Results indicated that the optimal temperature for the culture of *O. nana*, with significant differences compared to the other treatments, was 28 ± 1 °C. Regarding salinity, the optimal level for the total development of the population was 25 ± 2 PSU, while to maximize the percentage of copepodites, the most appropriate levels were 30 and 35 PSU. The light-dark regimen of 16L:8D promoted the highest total density of individuals, while 12L:12D and 16L:8D favored development of nauplii and copepodites. Finally, optimal concentrations of *I. galbana* that generated the highest densities of *O. nana* at all stages of its life cycle were 15 × 10^4^ and 20 × 10^4^ cells/mL. These findings provide key information for the development of optimized cultures of O. nana, which can improve its availability as a food source in aquaculture systems and favor the success of larval cultures in species of commercial interest.

## Simple Summary

This study determined the optimal culture conditions for the copepod *Oithona nana* to enhance its growth in aquaculture. The effects of temperature, salinity, photoperiod, and microalgal concentration were evaluated. The results indicated that the optimal temperature was 28 °C, salinity 25 PSU, and a photoperiod of 16L:8D. The best microalgal concentrations were 15 × 10^4^ and 20 × 10^4^ cells/mL, promoting higher densities across all life stages. These findings provide valuable information to improve the large-scale production of *O. nana*, supporting its use as a key food source in aquaculture and larval cultures for commercially important species.

## 1. Introduction

Zooplankton, composed of a wide variety of microscopic organisms, plays a crucial role in aquatic food webs, acting as a bridge between primary producers and higher trophic levels [1,2,3]. Within this group, copepods have been recognized for their ecological relevance and potential applicability in aquaculture, given their high nutritional quality and ability to support the larval growth of various commercially important marine species [4,5]. In particular, *Oithona nana*, a cyclopoid copepod widely distributed in marine and estuarine ecosystems [6], has attracted attention due to its remarkable resistance to environmental fluctuations, making it an ideal candidate for use as a live food source in aquaculture cultures [7].

Success of *O. nana* cultivation is strongly influenced by a variety of environmental and nutritional factors that affect its physiology, growth rate, and reproduction [7]. Among these factors, salinity, temperature, photoperiod, and diet play a key role, significantly influencing its development and survival under controlled conditions [7]. Several studies have demonstrated that copepods exhibit considerable phenotypic plasticity in response to changes in these parameters [8], highlighting the need to identify and optimize cultivation conditions to maximize their productivity and efficiency in aquaculture systems [9].

Temperature is a fundamental environmental parameter that directly influences metabolic rate [10,11,12], ontogenetic development [13,14], and reproductive efficiency in copepods [15,16]. It has been documented that different copepod species respond differently to thermal variations, with each species having an optimal temperature range for growth and reproduction [17,18,19]. For *O. nana*, determining the optimal temperature for cultivation is crucial for establishing efficient production protocols. Previous research has suggested that elevated temperatures may accelerate larval development [20,21], although at the cost of reduced longevity [22] and increased mortality rates [21], while lower temperatures can slow metabolism, leading to decreased growth and reproduction rates [23,24].

Salinity also influences osmotic balance [25], metabolism, and feeding efficiency in these species [26]. *O. nana* exhibits remarkable tolerance to a wide range of salinities, enabling it to inhabit both marine and estuarine waters [27]. However, optimizing salinity levels in controlled systems is essential to achieve optimal growth rates and maximize population biomass [28]. Abrupt fluctuations in salinity levels can induce physiological stress [29], affecting reproductive efficiency and copepod viability [30,31]. Recent studies have indicated that cyclopoid copepod species exhibit greater egg production and better hatching success when cultured under stable salinity conditions [32,33,34,35].

Photoperiod, defined as the alternating cycle between periods of light and darkness, also plays a crucial role in regulating the biological rhythms of copepods, influencing feeding [31,36,37], locomotion [38,39], and reproduction [40,41]. In planktonic organisms, light regulates these behaviors, which has direct implications for the mass production of *O. nana* [7]. Manipulating the photoperiod has proven to be an effective tool for increasing copepod productivity in cultures, as evidenced in other copepod species [42,43]. Prolonged exposure to light may alter swimming patterns [44], reducing feeding efficiency [45,46], while well-defined light-dark cycles promote synchronization of reproductive and molting cycles [43,47].

Diet is another essential factor regulating the growth and survival of *O. nana* [28,48]. The quality and availability of food significantly impact the reproduction rate and biochemical composition of its biomass [49,50]. Previous research has demonstrated that microalgae-enriched diets, especially those rich in essential fatty acids like DHA and EPA, improve copepod viability [50,51] and optimize their nutritional value as a live food source for aquaculture species [51,52]. Additionally, the combination of microalgae and symbiotic bacteria has been shown to improve feed conversion and digestibility in *O. nana*.

Despite significant advances in the biology and ecology of *O. nana*, knowledge gaps remain regarding how the combined interaction of these environmental factors affects its growth and survival. This study aims to evaluate the impact of salinity, temperature, photoperiod, and diet with *Isochrysis galbana* on the growth of *O. nana*, in order to generate key information that will enable the optimization of cultivation conditions for this copepod in aquaculture systems. *I. galbana* was chosen due to its high nutritional value, particularly its rich content of essential fatty acids such as DHA and EPA, which are critical for the growth, reproduction, and overall health of copepods. Its proven effectiveness in improving copepod viability and productivity, as well as its easy cultivation and stable nutritional profile, make it an ideal food source for *O. nana* in aquaculture systems. The results obtained will contribute to improving cultivation strategies, increasing copepod production in controlled environments, and facilitating the production of live feed for commercially important fish and crustacean species.

## 2. Materials and Methods

The experimental test was conducted at the Vila Vila Coastal Marine Laboratory (18°06′55.89″ S and 70°43′39.82″ W) of the Jorge Basadre Grohmann National University (UNJBG), located at km 61 of the Tacna—Ilo Coastal Highway, in the town of Vilavila, district of Sama—Las Yaras, province, and region of Tacna.

### 2.1. Biological Material

The copepod *O. nana* used in the experiment was provided by the Vila Vila Coastal Marine Laboratory. The maintenance conditions for the *O. nana* strain included: Temperature of 28 ± 0.5 °C, salinity of 35 PSU, photoperiod of 12L:12D, feeding with *Isochrysis galbana* (1 × 10^5^ cells/mL), light aeration, and irradiance of 40 µmol photon m^−2^ s^−1^. The microalga *I. galbana* was cultured using Guillard’s F/2 medium, and it was used during the exponential growth phase.

### 2.2. Experimental Trials

Before each experimental trial, the copepods were acclimated to the target salinities and temperatures for a period of 48 h. The acclimatization was carried out in 800 mL flasks, under controlled conditions (temperature 28 °C, salinity 35 PSU and photoperiod 12L:12D, feeding with 1 × 10^5^  *I. galbana*), ensuring that the copepods were fully adapted to the experimental environment. This acclimatization period was essential to ensure reliable results by minimizing the potential effects of environmental stress on the copepods’ behavior and development during the experiments. The cell counts and density adjustment of *I. galbana* were performed every 24 h to ensure adequate feeding density.

The study consisted of four experiments divided into two phases (Table 1):

Phase I: Determination of the optimal physical parameters (temperature, salinity, and photoperiod) for *O. nana* cultivation.

Phase II: Determination of the optimal microalgal density of *Isochrysis galbana* for *O. nana* cultivation, applying the optimal physical parameters.

All experiments were conducted under identical maintenance conditions for *Oithona nana*, using 1000 mL flasks containing 800 mL of microfiltered seawater (0.45 µm) and 10 ovigerous females in their first sexual maturity, selected under a stereoscope. Each experimental treatment was replicated four times to ensure the reliability and statistical power of the results. Population assessments were performed after 15 days of cultivation for each experiment, following the methodology outlined by Magouz et al. [28]. These conditions ensured consistency across replicates, minimized potential environmental variation, and allowed for reliable evaluations of copepod growth and reproductive performance.

#### 2.2.1. Experiment 1: Temperature

Four temperature treatments (20, 24, 28, and 32 °C) were evaluated with four replicates. The flasks were placed in isothermal boxes with thermostats (mark: SOBO, model: HS-100; Zhongshan Sobo Electrical Appliance Co., Ltd., Guangdong, China) and diffusing stones. Temperature was monitored twice a day and adjusted as necessary.

#### 2.2.2. Experiment 2: Salinity

Four salinity treatments (20, 25, 30, and 35 PSU) were evaluated with four replicates. Salinity was adjusted using seawater and distilled water according to the concentration equation (C1V1 = C2V2). It was checked bi-daily with a refractometer (±1 PSU, mark: ATAGO, model: 3810 PAL-1; ATAGO Co., Ltd, Tokio, Japan) and adjusted as needed.

#### 2.2.3. Experiment 3: Photoperiod

Three photoperiod treatments (12L:12D, 16L:8D, and 24L:0D) were evaluated with four replicates. Each treatment was controlled using 40-watt fluorescent lamps (mark: Philips, model: F40T12/D-765; Koninklijke Philips N.V., Amsterdam, The Netherlands) controlled by a timer (mark: Viox, model: THC-30A; Yueqing Viox Electric Co., Ltd., Yueqing, China).

#### 2.2.4. Experiment 4: Microalgal Density

Five densities of *I. galbana* (1 × 10^4^, 5 × 10^4^, 10 × 10^4^, 15 × 10^4^, and 20 × 10^4^ cells/mL) were evaluated with four replicates. The microalgal density was determined according to Paniagua et al. [53] and adjusted daily through counts in a Neubauer chamber.

### 2.3. Determination of Growth and Population Composition

After 15 days, the growth and population composition of the copepods were evaluated through the total harvest of the flasks. The samples were filtered using a 20 µm sieve and preserved in glass jars with seawater and 2% formalin (7.6 mL of seawater + 0.4 mL of 40% formalin).

For counting and identification, gridded Petri dishes were used under a stereomicroscope (mark: ZEISS, model: Stemi 305, Jena, Germany). The nauplii, copepodites, and adults (ovigerous females and males) were identified following the criteria of Takahashi and Uchiyama [54] and Ramírez and Derisio [55]. The counts were performed in triplicate.

### 2.4. Data Analysis

Statistical analyses were performed using the Rstudio statistical software version 2024.04.2 + 764 from Rstudio, Inc. (Boston, MA, USA). All data were subjected to normality tests using the Anderson-Darling test and variance homogeneity using Bartlett’s test. Data were evaluated with one-way analysis of variance (ANOVA) and Tukey’s post hoc test. When assumptions were not met, data were analyzed using the Kruskal–Wallis test to assess differences between treatments and the Dunnett test to determine homogeneous groups [56]. A principal component analysis (PCA) was performed to evaluate the relationship between total population and culture parameters. Graphs were made using the ggplot2 package of Rstudio and expressed as mean ± standard deviation (SD).

## 3. Results

### 3.1. Temperature

**Figure 1:**
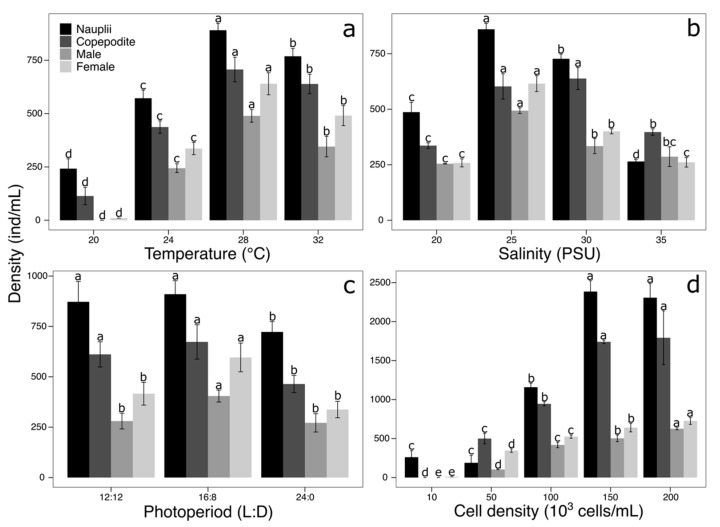
Density of *Oithona nana* by stage at the end of the experiment (**a**) Temperature, (**b**) salinity, (**c**) Photoperiod and (**d**) *Isochrysis galbana* cell density. The letters above each bar indicate statistically significant differences between treatments.

The nauplii and adult copepods showed a normal distribution (*p* > 0.05), but this was not the case for the copepodites (A = 0.737, *p* = 0.043) and total population (A = 0.750, *p* = 0.040). All data were influenced by the culture temperature (ANOVA, *p* < 0.05) (Figure 1a). The highest total population density was reached at 28 °C (3408 ± 80.71 ind/mL), followed by 32 °C (2805 ± 76.22 ind/mL). At 24 °C, the density was intermediate (1970 ± 66.82 ind/mL), and the lowest density was recorded at 20 °C (457 ± 70.47 ind/mL) (Table 2). The total population showed significant differences among all treatments (K − W = 14.118, *p* < 0.01), especially between 28 °C and the others, identifying this value as optimal for cultivation, with a specific growth rate (SGR) of 0.33 d^−1^ and a sex ratio of 0.77 (Table 2).

### 3.2. Salinity

Except for the nauplii (A = 0.631, *p* = 0.081), densities in the salinity treatments did not follow a normal distribution (*p* < 0.05). Highly significant differences were found between treatments (*p* < 0.01) (Figure 1b). The highest total population was obtained at 25 PSU (3214 ± 48.60 ind/mL), followed by 30 PSU (2625 ± 42.57 ind/mL), while the lowest was at 35 PSU (1512 ± 29.88 ind/mL). The total population showed significant differences between all treatments (K − W = 13.787, *p* < 0.001). The optimal salinity for population growth was 25 PSU, with an SGR of 0.32 d^−1^, and a sex ratio of 0.73.

### 3.3. Photoperiod

Normality (*p* > 0.05) and homogeneity of variances (*p* > 0.05) were confirmed for all developmental stages and the total population. The densities in the *O. nana* treatments showed significant differences between photoperiod treatments (ANOVA, *p* < 0.01) (Figure 1c). The 16L:8D photoperiod produced the highest density (3230 ± 62.28 ind/mL), followed by 12L:12D (2970 ± 56.14 ind/mL). The lowest density was recorded in the continuous light treatment (24L:0D) with 2245 ± 85.87 ind/mL. Post hoc analysis revealed that 16L:8D was significantly higher than the other treatments (*p* < 0.05), with an SGR of 0.34 d^−1^ and a sex ratio of 0.81 (Table 2).

### 3.4. Microalgal Density

The densities of *O. nana* by stage did not meet the normality assumption (*p* < 0.05), except for the copepodites (A = 0.688, *p* = 0.061). The concentration of *I. galbana* also had a highly significant effect on the total density of *O. nana* (K − W, *p* < 0.01) (Figure 1d). The highest densities were recorded at 150 × 10^3^ cells/mL (6588 ± 154.29 ind/mL) and 200 × 10^3^ cells/mL (6816 ± 239.83 ind/mL), while the lowest densities corresponded to 10 × 10^3^ and 25 × 10^3^ cells/mL, both below 2500 ind/mL. The total population analysis indicated significant differences between groups with low and high food availability (A = 17.6, *p* < 0.01), establishing an optimal threshold starting at 150 × 10^3^ cells/mL, with the best growth rate and a more balanced proportion of male and female individuals, resulting in a sex ratio of 0.86.

### 3.5. Multivariate Analysis

**Figure 2:**
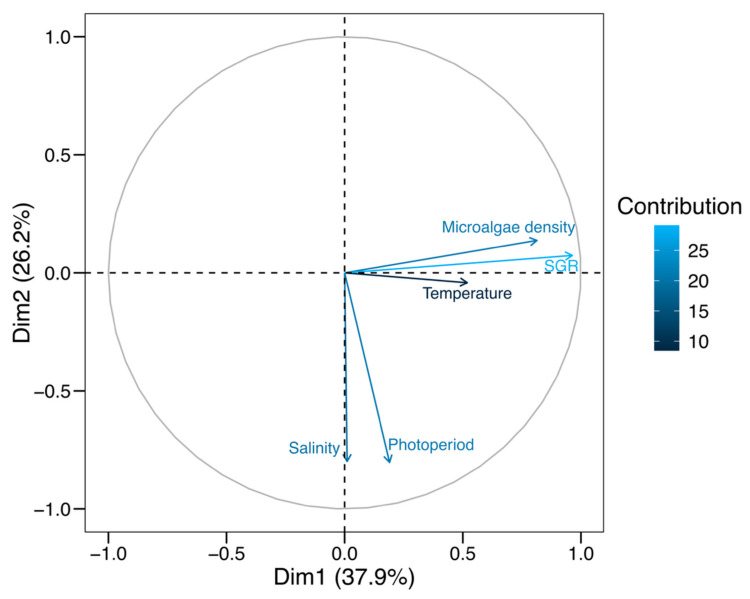
Principal component analysis (PCA) of experimental culture parameters and specific growth rate (SGR) of *Oithona nana*. The circle highlights the cluster of data points with high correlation between the experimental parameters.

The principal component analysis (PCA) conducted on the experimental cultivation parameters of *O. nana* (temperature, salinity, photoperiod, microalgae density, and specific growth rate index, SGR) revealed a notable separation of the influencing factors across the first two PCA dimensions (Dim1: 37.9%, Dim2: 26.2%), together explaining 64.1% of the data variability (Figure 2). The PCA vectors indicated a strong positive association between microalgae density and SGR, suggesting that greater food availability could promote an increase in the growth rate of *O. nana*.

The correlation between temperature and SGR was moderate and positive (0.4511), suggesting that an increase in temperature is associated with a concomitant increase in the specific growth rate, although the relationship is not particularly strong. In contrast, the correlation between salinity and SGR was negligible and negative (−0.0089), indicating that salinity has a minimal effect on the specific growth rate under the experimental conditions. The correlation between photoperiod and SGR was low and positive (0.0856), implying that the photoperiod has a weak, though positive, influence on SGR, potentially contributing little to its variability. Conversely, the correlation between cell density and SGR was strong and positive (0.7531), highlighting a significant positive relationship, whereby an increased number of cells is strongly associated with a higher SGR.

## 4. Discussion

Optimizing the cultivation conditions of aquatic organisms, such as copepods, is a critical step in the development of sustainable aquaculture systems. *O. nana*, a key species in marine aquaculture due to its role in trophic webs and its high nutritional quality as a live food source, is sensitive to a variety of environmental factors.

### 4.1. Temperature

Temperature is a crucial factor in the physiology of copepods, as it directly influences metabolic rate, development, and reproduction of these organisms [21,24]. In this study, the optimal temperature for *O. nana* growth was found to be 28 °C, which is consistent with the findings of Huanacuni et al. [7], who reported that *Oithona* and other marine copepod species exhibit higher growth and reproduction rates at temperatures close to 28 °C [57,58]. This phenomenon occurs because, within this thermal range, copepods can maximize their food conversion rate without suffering the adverse effects of thermal stress. On the other hand, at temperatures above 30 °C, copepods experienced a decrease in population density, which may be explained by thermal stress [19,24]. Liu and Ban [59] and Rueda Moreno and Sasaki [60] observed that high temperatures accelerate metabolic processes in copepods, which can lead to faster depletion of their energy reserves and, consequently, increased mortality. Additionally, high temperatures can destabilize the ionic balance of copepods, compromise their homeostasis and affect their viability [11,24]. This finding is consistent with the results of Vu et al. [16], who documented that marine copepod species show a decrease in their reproduction and growth rates at temperatures above 30 °C due to thermal stress. These negative effects are especially relevant in copepod cultures, where temperature conditions must be carefully controlled to avoid premature mortality and low production yields [19,61]. In contrast, at 20 °C, *O. nana* growth was considerably slower [25,62]. This phenomenon can be explained by the reduction in metabolic rate at low temperatures, which slows down copepod development and reduces food conversion efficiency [11]. Copepod species, including *O. nana*, depend on moderate temperatures to maintain an efficient metabolism that favors their growth and reproduction.

This study also demonstrated that temperature has a considerable impact on the male-to-female ratio and SGR, reinforcing the notion that thermal control is essential for maintaining a balanced and productive population. At 28 °C, the sex ratio was close to equilibrium (0.77), favoring reproductive stability. However, at 32 °C, the sex ratio skewed towards females (0.71), which could indicate an increase in female fecundity or greater resistance of females to extreme thermal conditions, as documented by previous studies [17,63]. This phenomenon of a higher female-to-male ratio at elevated temperatures could be explained by a higher energy requirement for males, which affects their survival and growth [60]. At the same time, the higher growth rate at 28 °C may be related to the optimization of metabolic processes and the greater availability of resources that allow for faster and more efficient development of individuals within this thermal range.

### 4.2. Salinity

Salinity significantly affects the metabolism of copepods, influencing osmotic balance [25,29,64] and the organisms’ ability to regulate ion concentration in their cells [65,66]. In this study, a salinity of 25 PSU produced the highest density of *O. nana*, which is consistent with other studies reporting that marine copepod species exhibit optimal growth at moderate salinities [67,68]. Magouz et al. [28] demonstrated that a salinity of 20 PSU favors the growth and reproduction rate of *O. nana*, suggesting that it is the optimal value that allows for osmotic balance without inducing physiological stress. At lower salinities, such as 20 PSU, *O. nana* copepods experienced slower growth, which could be the result of osmotic stress, as copepods need to expend more energy to regulate their internal ion concentration [69]. Uye and Sano [70] reported that estuarine copepod species like *O. nana* have greater tolerance to salinity fluctuations but still require a moderate salinity range to maintain optimal growth [28]. At lower salinities, copepods may face difficulties in maintaining homeostasis, resulting in slower growth rates and higher mortality [28,71]. As for higher salinities (35 PSU), the results indicated that *O. nana* also experienced a decrease in population density. This phenomenon could be explained by osmotic stress induced by high salinity, which may affect the copepods’ reproductive efficiency and their ability to efficiently absorb nutrients [72]. The increased difficulty in regulating ionic balance at high salinities may have reduced the viability of the copepods, leading to a decrease in their growth.

### 4.3. Photoperiod

Photoperiod is an important factor in the regulation of biological and reproductive rhythms in copepods, as it influences their swimming behavior [73,74], feeding [36,75], and reproduction [42,47]. In this study, the 16L:8D photoperiod produced the highest density of *O. nana*, which is consistent with the literature suggesting that a well-defined light-dark cycle favors the synchronization of reproductive cycles [31] and feeding efficiency in copepods [76]. Camus and Zeng [43] demonstrated that a 16L:8D photoperiod optimizes copepod growth rate by facilitating the synchronization of egg release and improving feeding efficiency. In contrast, the 24L:0D photoperiod, representing continuous light exposure, resulted in the lowest population density, suggesting that constant exposure to light may disrupt copepod circadian rhythms, reducing their reproductive and feeding efficiency [40,42]. This finding agrees with the results of Matias-Peralta et al. [15], who found that continuous light negatively affects copepod reproduction rates and alters their swimming and feeding cycles. This effect is likely due to the dysregulation of circadian cycles, which copepods use to coordinate their feeding and reproductive behaviors. Disruption of these cycles may result in decreased feeding efficiency and fewer eggs produced, which reduces population density [63]. Camus and Zeng [43] also observed that a well-defined photoperiod, such as 16L:8D, is essential to maximize productivity in copepod cultures.

The photoperiod played an important role in maximizing population density, especially in the 16L:8D treatment, which produced the highest density and an SGR of 0.34 d^−1^. These results are consistent with previous studies suggesting that an appropriate photoperiod facilitates the synchronization of reproductive cycles and enhances feeding efficiency, resulting in an increase in growth rate [31,42]. The male-to-female ratio in this photoperiod was favorable, with a sex ratio close to 0.68, reflecting an environment conducive to balanced reproduction.

### 4.4. Microalgal Density

The feed conversion efficiency (FCR) of copepods is directly related to the quantity and quality of microalgae available in the water [77]. When the microalgae density is adequate, copepods can maximize food conversion and use these resources to increase their body mass, which in turn reflects an increase in population density [78,79]. This phenomenon is observed in several copepod species, such as *Acartia tonsa* and *Calanus finmarchicus*, whose growth and reproduction rates are enhanced with greater availability of microalgae [80]. In the case of *O. nana*, the highest density observed at higher microalgae concentrations (15 × 10^4^ and 20 × 10^4^ cells/mL) can be explained by greater nutrient availability for the copepods [48,81]. When copepods have access to sufficient nutrients, they can maintain a favorable metabolic balance that optimizes their growth and reproduction [24]. Furthermore, smaller copepod species, such as *O. nana*, generally have higher efficiency in converting microalgae to biomass due to their size and high nutritional value [51,82]. The availability of microalgae rich in essential fatty acids is also critical, as these compounds are essential for cellular membrane synthesis, neuronal function, and egg production in copepods [52]. In contrast, lower concentrations of microalgae (1 × 10^4^ and 5 × 10^4^ cells/mL) led to a lower population density of *O. nana*, suggesting that the amount of food available was insufficient to meet the metabolic demands of the copepods [83,84]. This phenomenon can be explained by the decreased feed conversion rate when copepods do not have sufficient microalgae availability to meet their energy and growth needs. The low microalgae density can also lead to a deficiency in essential nutrients, such as Omega-3 fatty acids (EPA and DHA), which are vital for copepod development [85,86,87]. The microalgae densities at 15 × 10^4^ and 20 × 10^4^ cells/mL produced the highest *O. nana* densities, which also resulted in a high SGR. This phenomenon is due to the greater nutrient availability, allowing for more efficient food conversion and higher reproduction rates [48]. The sex ratio in these treatments remained balanced, highlighting the importance of an adequate diet for maintaining population stability.

### 4.5. Multivariate Analysis

Results obtained in this study reinforce the idea that optimizing *O. nana* cultivation conditions requires a multifactorial approach, in which temperature and microalgae density play prominent roles. The PCA highlighted that microalgae density has a stronger correlation with specific growth rate (SGR), supporting previous findings that indicate that high concentrations of phytoplankton improve copepod viability and development [51,52]. The optimal temperature of 28 °C identified in this study is consistent with the literature on marine copepods, where it has been reported that *Oithona* species experience more efficient growth within moderate thermal ranges, without reaching thermal limits that could induce stress [7,82]. On the other hand, the less significant effects of salinity and photoperiod observed in this study align with previous research suggesting that salinity may have an indirect influence on *O. nana* growth by altering its osmotic balance [28]. However, the optimal salinity identified in this study (25 PSU) is consistent with the range found for other estuarine copepods, which require moderate conditions to maximize their development [32]. Furthermore, although the importance of photoperiod was less prominent in terms of correlation with growth, it cannot be underestimated given its role in regulating the biological and reproductive rhythms of copepods, as evidenced in studies on other tropical copepod species [42,43]. The results suggest that under controlled conditions, a photoperiod of 16L:8D is ideal for maximizing population density, and that manipulation of light and dark cycles can have a significant impact on productivity in mass cultures of *O. nana*.

In addition to the environmental parameters tested in this study, the El Niño-Southern Oscillation (ENSO) phenomenon, which is known to influence oceanographic conditions, can also affect zooplankton activity and population dynamics [88]. ENSO events, characterized by shifts in sea surface temperature and ocean currents, may lead to significant changes in the abundance, distribution, and reproductive success of marine zooplankton, including copepods [88,89]. Although our study was conducted under controlled laboratory conditions, it is important to acknowledge that natural fluctuations in temperature and salinity, which are often associated with ENSO events, could impact the productivity of *O. nana*. Our results, showing optimal conditions for temperature and salinity, align with those typically observed during periods of environmental stability, suggesting that ENSO-induced variations may influence copepod growth and reproduction. Future studies could explore the interaction between ENSO and copepod cultivation to further optimize production protocols under varying environmental conditions [88].

Together, these results provide a deeper understanding of how to optimize cultivation conditions for *O. nana*, which may have practical implications for its use as a live food source in aquaculture systems. Despite advancements in copepod cultivation, further studies are needed to explore the complex interactions between environmental factors and their impact on the different life stages of *O. nana* to improve production efficiency and the sustainability of aquaculture cultures.

## 5. Conclusions

This study provides crucial information for optimizing the culture conditions of *O. nana*, a copepod that is essential in aquaculture due to its value as a source of live feed. The results show that a temperature of 28 °C and a microalgae density of 15 × 10^4^ to 20 × 10^4^ cells/mL are the most decisive factors in the growth and population density of *O. nana*. In terms of salinity, 25 PSU was found to favor overall population growth, while salinities of 30 and 35 PSU were more effective in increasing the proportion of copepodites. The 16L:8D photoperiod proved to be the most efficient for maximizing individual density, highlighting the importance of a well-regulated light and dark cycle. Although salinity and photoperiod had a less significant impact than temperature and food availability, their influence should not be underestimated in the cultivation optimization process. These findings provide key parameters for improving copepod production in aquaculture systems, and further research is needed to better understand the interactions between these factors under large-scale cultivation conditions, moving aquaculture towards a more efficient and sustainable model.

## Figures and Tables

**Table 1:** Experimental cultivation parameters of *Oithona nana*.

Variable	Stage I			Stage 2
	Experiment 1 (Temperature)	Experiment 2 (Salinity)	Experiment 3 (Photoperiod)	Experiment 4 (Microalgal Density)
Temperature (°C)	T1: 20	28	28	28
	T2: 24			
	T3: 28			
	T4: 32			
Salinity (PSU)	35	T1: 20	35	25
		T2: 25		
		T3: 30		
		T4: 35		
Photoperiod (L:D)	12:12	12:12	T1: 12:12	16:8
			T2: 16:8	
			T3: 24:0	
Microalgal density (cells/mL)	1 × 10^5^	1 × 10^5^	1 × 10^5^	T1: 1 × 10^4^
				T2: 5 × 10^4^
				T3: 10 × 10^4^
				T4: 15 × 10^4^
				T5: 20 × 10^4^

**Table 2:** Biological parameters of *Oithona nana* under experimental conditions.

Biological Parameters	Treatment	Experiment			
		Temperature (°C)	Salinity (PSU)	Photoperiod (L:D)	Microalgal Density (Cells/mL)
Total population (ind)	T1	457 ± 70.47 ^d^	1670 ± 59.10 ^c^	2725 ± 145.39 ^b^	339 ± 72.15 ^d^
	T2	1987 ± 32.65 ^c^	3214 ± 48.60 ^a^	3230 ± 62.28 ^a^	1423 ± 90.17 ^c^
	T3	3408 ± 80.71 ^a^	2625 ± 42.57 ^b^	2245 ± 85.87 ^c^	3809 ± 34.09 ^b^
	T4	2805 ± 76.22 ^b^	1512 ± 29.88 ^d^		6588 ± 154.29 ^a^
	T5				6816 ± 239.83 ^a^
Sex ratio (M/F)	T1	0.00 ± 0.00 ^b^*	0.99 ± 0.06 ^ab^	0.68 ± 0.10 ^a^	0.00 ± 0.00 ^c^*
	T2	0.73 ± 0.06 ^a^	0.81 ± 0.06 ^a^	0.68 ± 0.05 ^a^	0.30 ± 0.02 ^b^
	T3	0.77 ± 0.02 ^a^	0.83 ± 0.06 ^ab^	0.81 ± 0.09 ^a^	0.80 ± 0.05 ^a^
	T4	0.71 ± 0.09 ^a^	1.11 ± 0.22 ^b^		0.79 ± 0.12 ^a^
	T5				0.86 ± 0.04 ^a^
SGR (d^−1^)	T1	0.20 ± 0.015 ^d^	0.28 ± 0.00 ^c^	0.31 ± 0.005 ^b^	0.17 ± 0.02 ^d^
	T2	0.29 ± 0.002 ^c^	0.32 ± 0.00 ^a^	0.32 ± 0.002 ^a^	0.27 ± 0.01 ^c^
	T3	0.33 ± 0.002 ^a^	0.31 ± 0.00 ^b^	0.30 ± 0.004 ^c^	0.34 ± 0.00 ^b^
	T4	0.32 ± 0.003 ^b^	0.27 ± 0.00 ^d^		0.37 ± 0.00 ^a^
	T5				0.37 ± 0.00 ^a^

* No presence of adult male copepods. Different superscript letters (a–d) across columns indicate statistically significant differences between treatments.

## Data Availability

The data presented in this study are available on request from the corresponding author.
